# Supplementary material for: Biomimetic high performance artificial muscle built on sacrificial coordination network and mechanical training process
Source: Nat Commun. 2021 May 18;12:2916. doi: 10.1038/s41467-021-23204-x (PMC8131361; doi:10.1038/s41467-021-23204-x)
Supplement: Supplementary file 1 — Supplementary Information [file 41467_2021_23204_MOESM1_ESM.pdf]

Supplementary Information

Biomimetic High Performance Artificial Muscle Built on Sacrificial Coordination Network and Mechanical Training Process

Zhikai Tu[1,4], Weifeng Liu[1,4]*, Jin Wang[2], Xueqing Qiu[3,]*, Jinhao Huang[1], Jinxing Li[1] & Hongming Lou[1]

[1] School of Chemistry and Chemical Engineering, Guangdong Engineering Research Center for Green Fine Chemicals, South China University of Technology, Guangzhou 510640, P. R. China.

[2] The National Engineering Research Center of Novel Equipment for Polymer Processing, School of Mechanical & Automotive Engineering, South China University of Technology, Guangzhou 510640, P. R. China.

[3] School of Chemical Engineering and Light Industry, Guangdong University of Technology, Guangzhou 510006, P. R. China.

[4] These authors contributed equally: Zhikai Tu, Weifeng Liu.

*email: weifengliu@scut.edu.cn (W. L.), qxq@gdut.edu.cn (X. Q.)

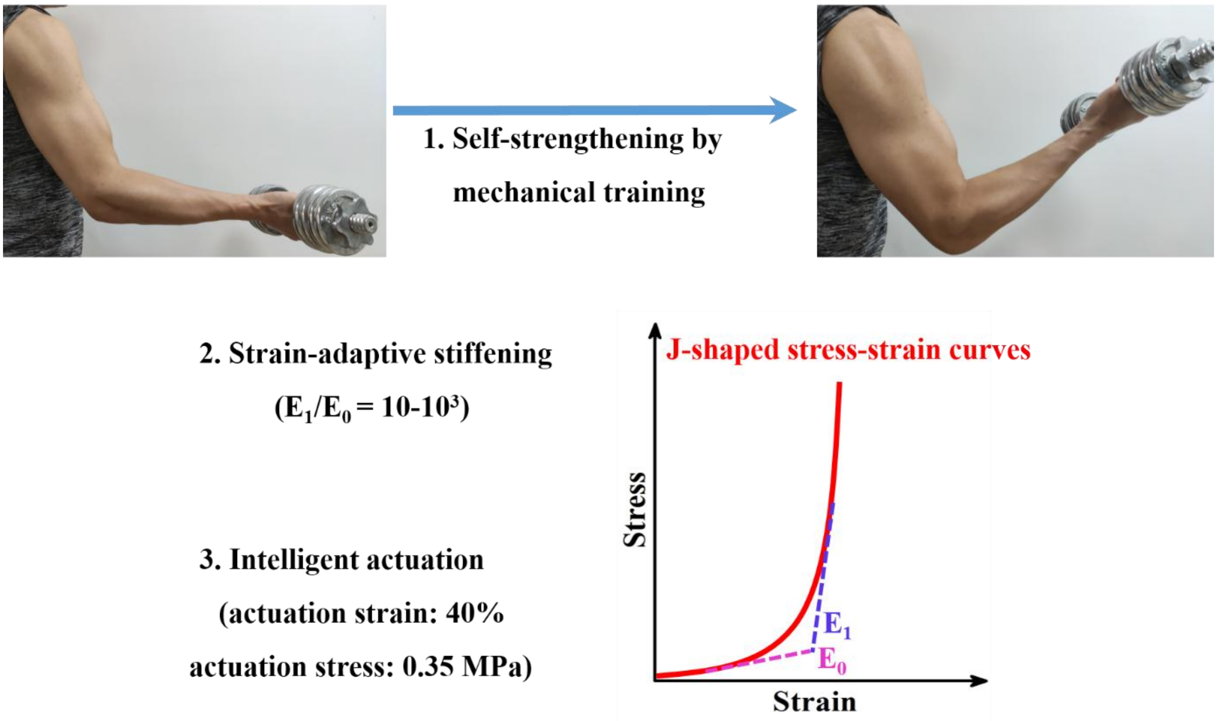

Supplementary Fig. 1 Characteristics of skeletal muscles[1-3].

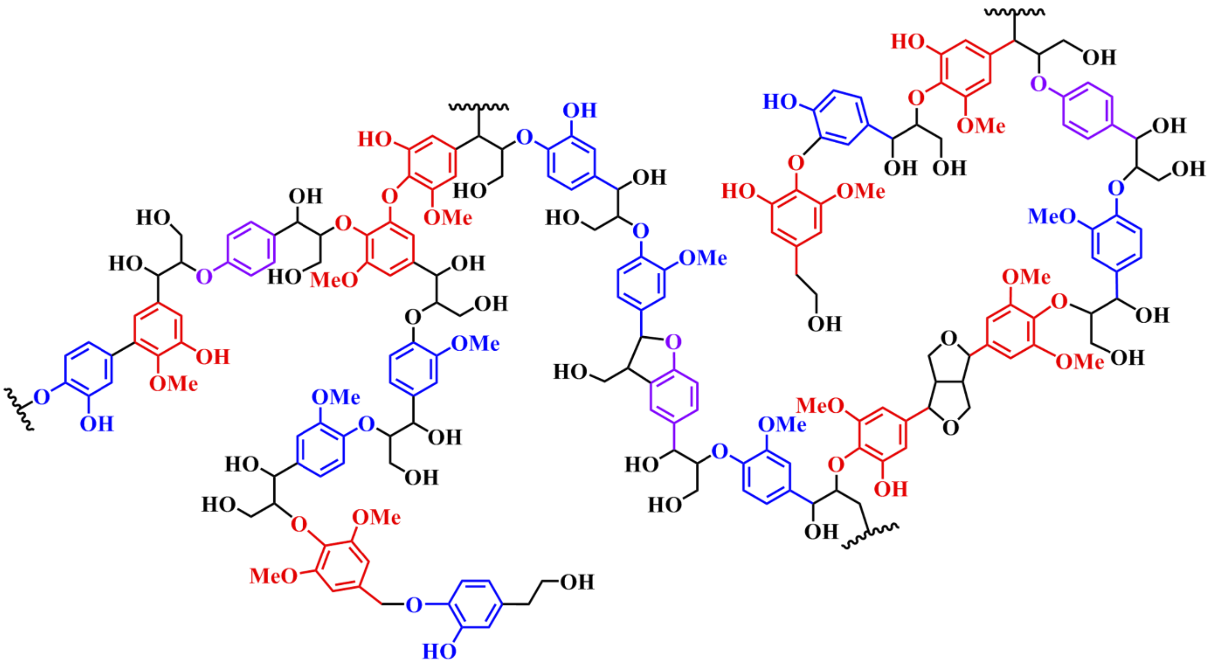

Supplementary Fig. 2 The chemical structure illustration of lignin.

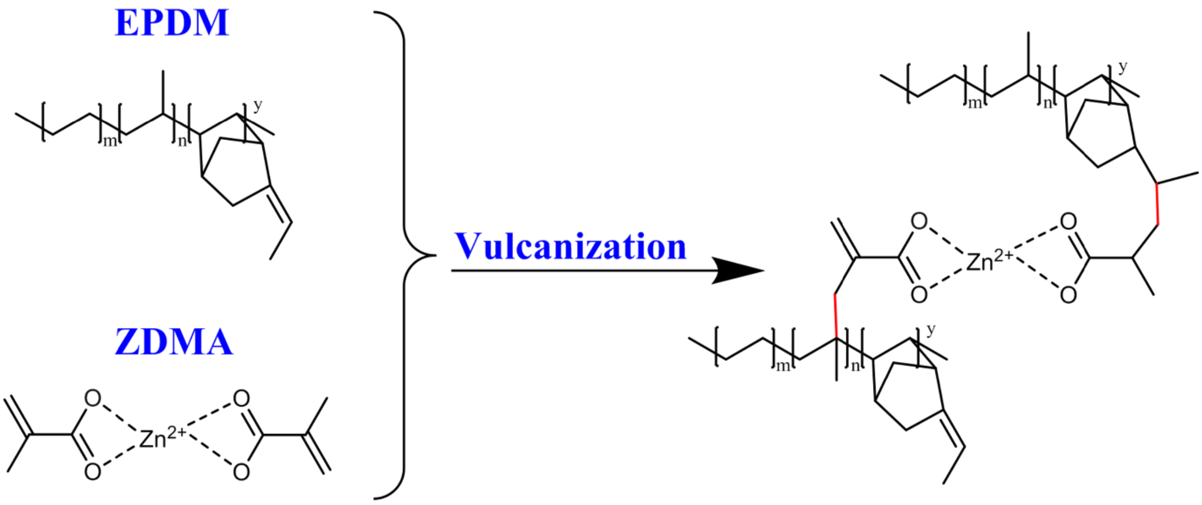

Supplementary Fig. 3 The reaction mechanism between EPDM and ZDMA. More detailed vulcanization mechanism of EPDM could be found in Reference [4] in this document.[4]

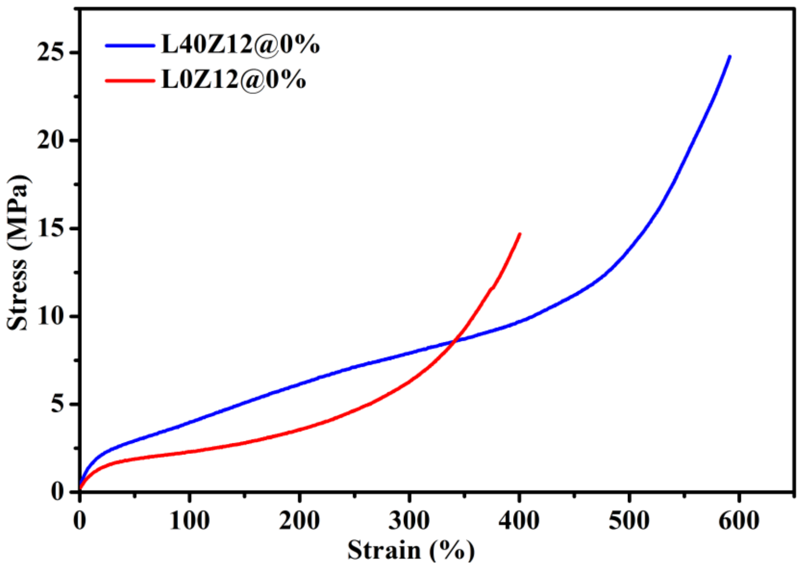

Supplementary Fig. 4 The engineering stress-strain curves of L40Z12@0% and L0Z12@0%.

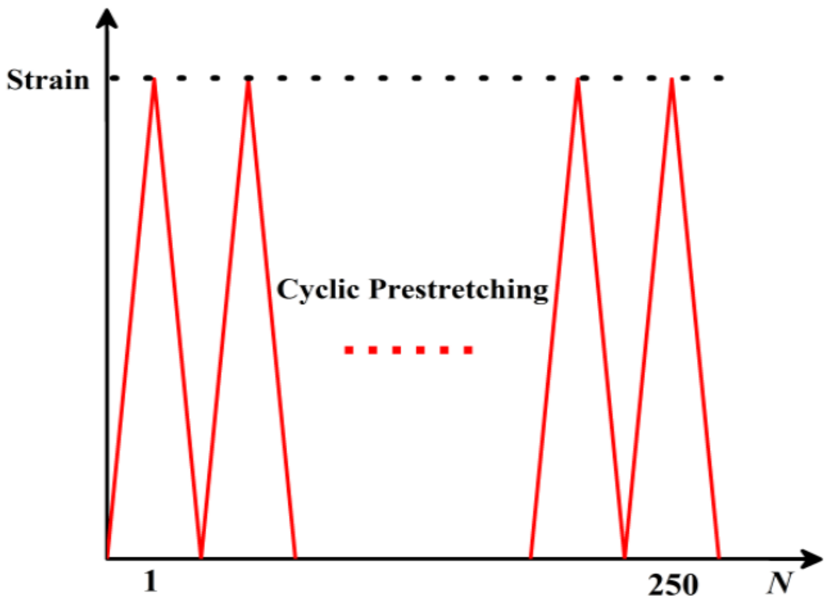

Supplementary Fig. 5 Illustration for the repeated pre-stretching of mechanical training process.

The key challenge for mechanical training was to undergo repeated and severe pre-stretching without causing the material failure. For the EPDM elastomer without coordination bonds (L40Z0@0%), a small initial fracture would rapidly extend to the failure of the whole material during the repetitive loading cycles at the critical strain. By contrast, L40Z12@0% containing the dynamic coordination bonds could dissipate external energy by rupture and reform, thus preventing the defect growth inside the material.

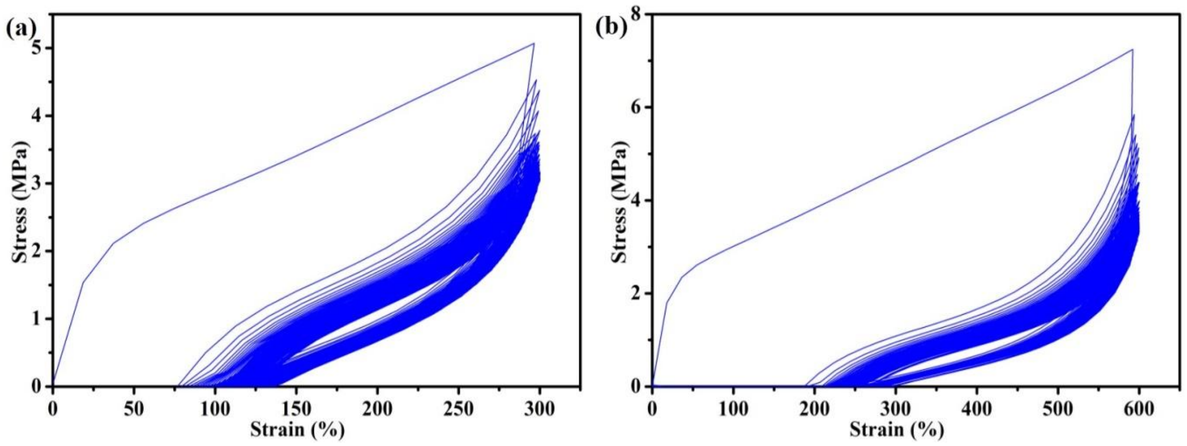

Supplementary Fig. 6 a) The loading-unloading curves of L40Z12@300% during mechanical training; b) The loading-unloading curves of L40Z12@600% during mechanical training.

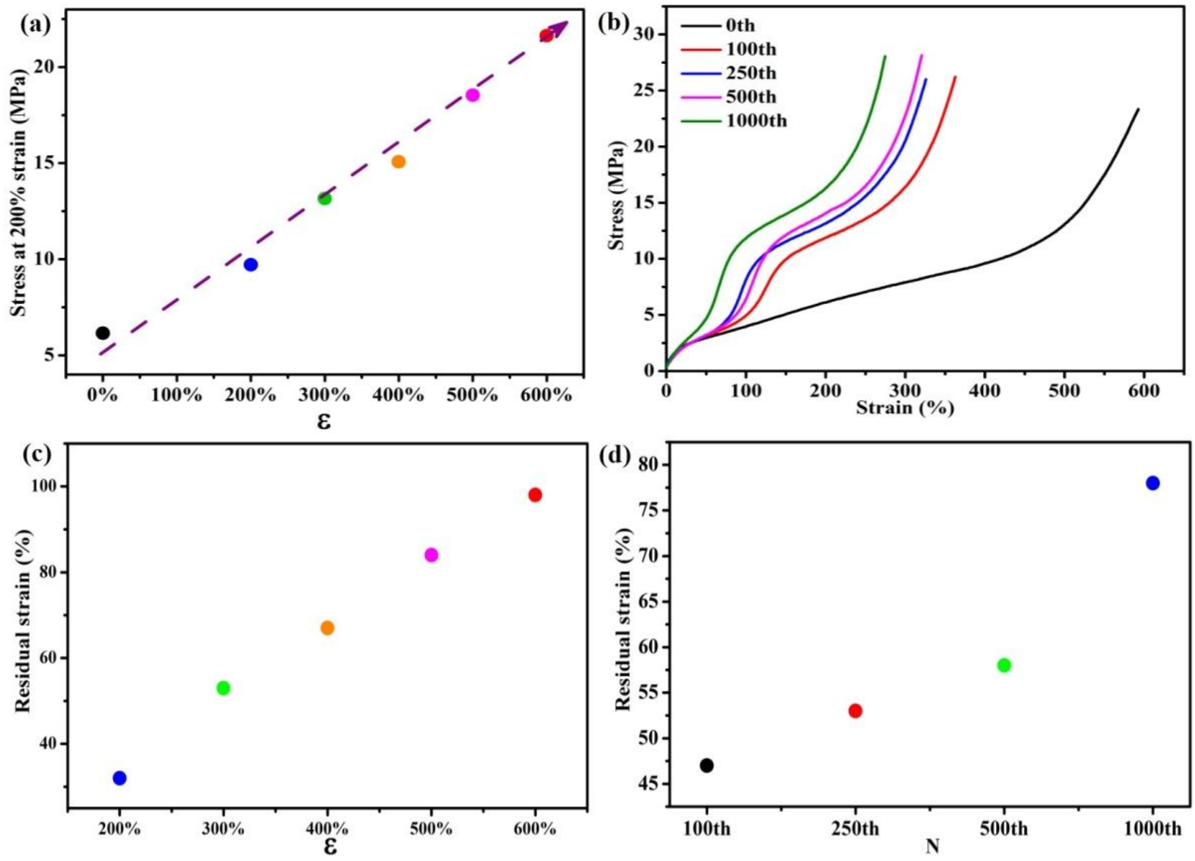

Supplementary Fig. 7 a) The stress at 200% strain of EPDM composites after mechanical training for 250 cyclyes at different training strain; b) The engineering stress-strain curves of EPDM composites after mechanical training at 300% training strain under different cycles; c) The residual strain of EPDM composites after mechanical training for 250 cyclyes under different training strain; d) The residual strain of EPDM composites after mechanical training at 300% training strain under different cycles.

Under the repeatitive prestretching of mechanical training, the residual strain of the elastomer gradually increased with the training strain and training frequency (Supplementary Figs. 7c, d), indicating that the chain alignment was stabilized by the coordination bonds in the elastomer composite L40Z12. The reason for this trend could be that, since the larger strain and frequency were applied in mechanical training, the more dynamic coordination bonds were involved in destruction and reconstruction course, and thus more chain alignments were stabilized along the prestretching direction after mechanical training.

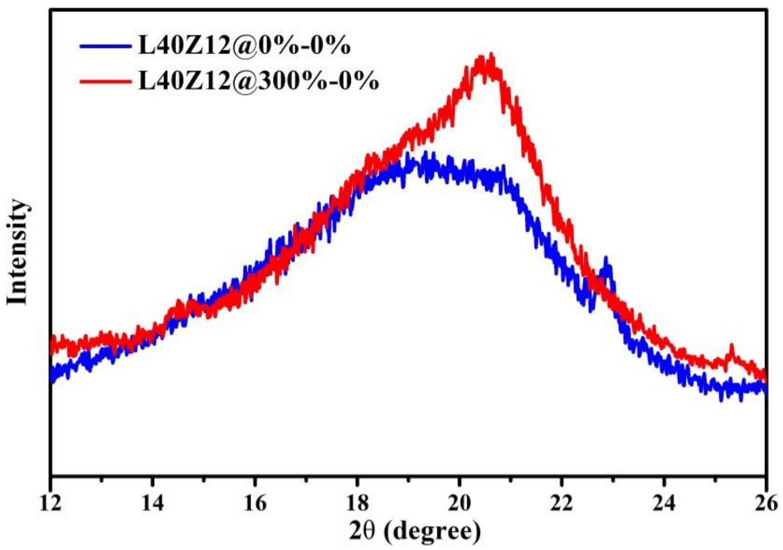

Supplementary Fig. 8 The XRD patterns of L40Z12@0% and L40Z12@300% at 0% strain.

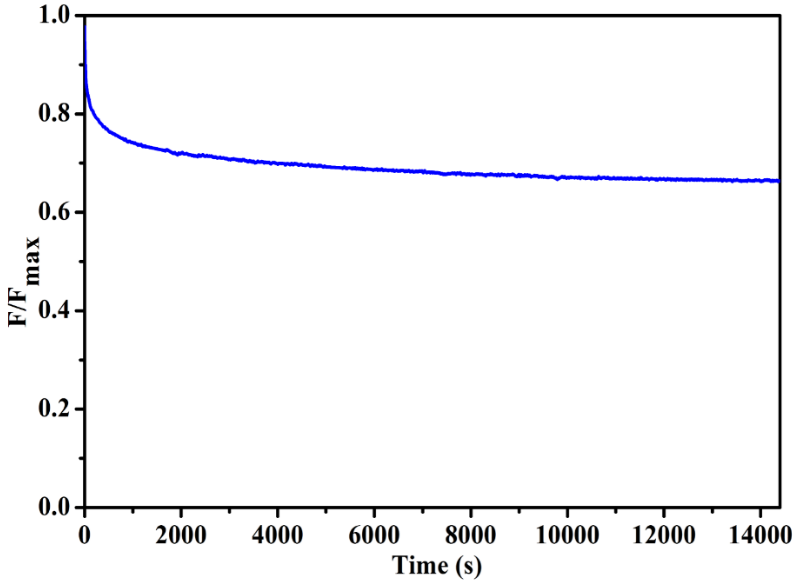

Supplementary Fig. 9 The stress relaxation curve of L40Z12@300% at 50% strain.

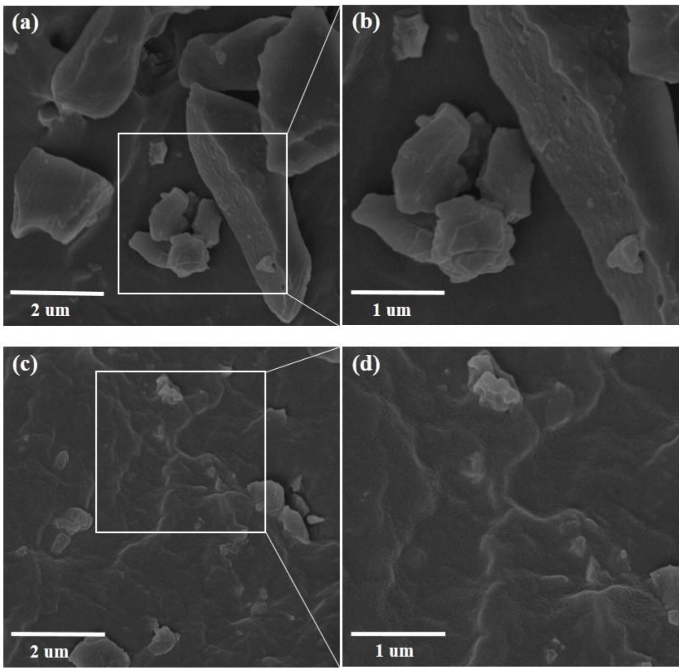

Supplementary Fig.10 a,b) SEM images taken from the cross sections of L40Z12@0% without mechanical training and c,d) L40Z12@600%.

To investigate the self-strengthening mechanism by mechanical training, the microphase separation of the sample L40Z12 before and after training was studied by SEM as shown in Supplementary Fig 10. Without the mechanical training process, the lignin agglomerates of micron level were observed in the sample L40Z12@0%, and the interface between the agglomerated lignin particles and polymer matrix was obviously exfoliated (Supplementary Figs. 10a, b). Nevertheless, after mechanical training, the lignin particle size was reduced without obvious large agglomerates in L40Z12@600%, and the interface became blurry and more cohesive (Supplementary Figs. 10c, d), suggesting improved interfacial interactions. As the dynamic coordination bonds could increase the interfacial interactions between lignin and polymer matrix, the variation in the phase-separation structure after mechanical training indicated that, the interfacial coordination bonds promoted the internal stress transfer from EPDM matrix to lignin particles, leading to repeated shearing on lignin particles during the mechanical training process, and thus resulting in smaller particle size and better dispersion of lignin in polymer matrix.

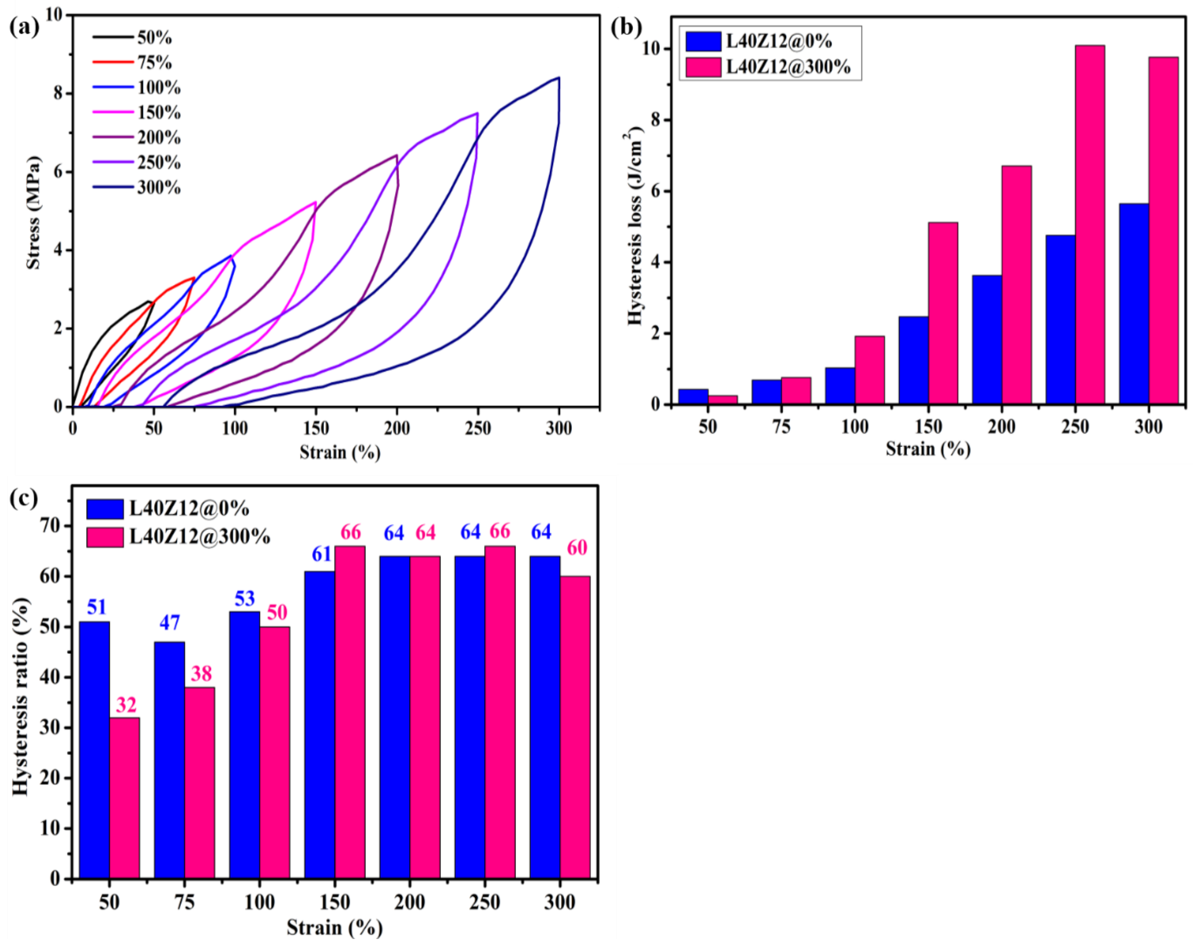

Supplementary Fig. 11 a**)** The hysteresis tensile curves of L40Z12@0% at various strains from 50% to 300%; b**)** The hysteresis loss of L40Z12@300% and L40Z12@0%; c**)** The hysteresis ratio of L40Z12@300% and L40Z12@0%.

For the sample of L40Z12@0% without mechanical training, the hysteresis ratio at 50% strain was higher than L40Z12@300% (Supplementary Figs. 11a-c), but increased slightly when the applied strain was between 75% and 150%, followed by stable value at strains larger than 150%. This suggested that, for L40Z12@0% without mechanical training, the dynamic fracture of coordination bonds might proceed continuously during the whole stretching process.

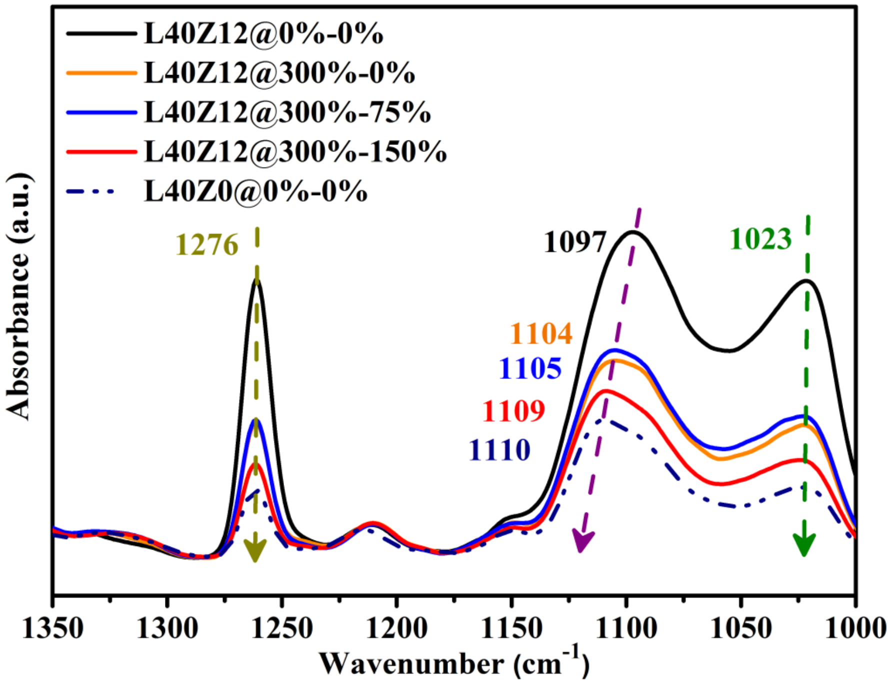

Supplementary Fig. 12 The FTIR spectra of L40Z12@300% at the tensile strain of 0%, 75% and 150%, in comparison with the samples L40Z12@0% and L40Z0@0% at the strain of 0% (a.u. means absorbance unit).

In order to substantiate the fracture of coordination bonds in the strain from 75% to 150%, the FTIR spectra of L40Z12@300% at the corresponding strains were examined. As shown in Supplementary Fig. 12, comparing with L40Z12@0%-0%, the signals of 1276 cm^{-1}, 1097 cm^{-1} and 1023 cm^{-1} corresponding to the coordination effect between Zn^{2+} and oxygen-containing groups in L40Z12@300%-0% all showed substantial decrease in intensity, accompanied by a blueshift from 1097 cm^{-1} to 1104 cm^{-1}, confirming that part of dynamic coordination bonds were fractured after the mechanical training process. When the sample L40Z12@300% was stretched to 75% strain, the FTIR spectrum of L40Z12@300%-75% was almost the same as that of L40Z12@300%-0%, suggesting that the state of coordination bonds in L40Z12@300% did not change obviously when the strain was less 75%. However, as the sample L40Z12@300% was further stretched to 150% strain, the peak intensity of 1276 cm^{-1}, 1097 cm^{-1} and 1023 cm^{-1} all evidently decreased, and a blueshift from 1105 cm^{-1} to

1109 cm^{-1} was also observed, showing a much weaker coordination bonding effect in L40Z12@300%-150% than in L40Z12@300%-75% and L40Z12@300%-0%. The FTIR spectrum of L40Z12@300%-150% was close to the sample of L40Z0@0%-0% without coordination bonds. These signal variations demonstrated that the dynamic coordination bonds in L40Z12@300% mainly fractured in the strain range from 75% to 150%, coincident with the results of the hysteresis tensile tests.

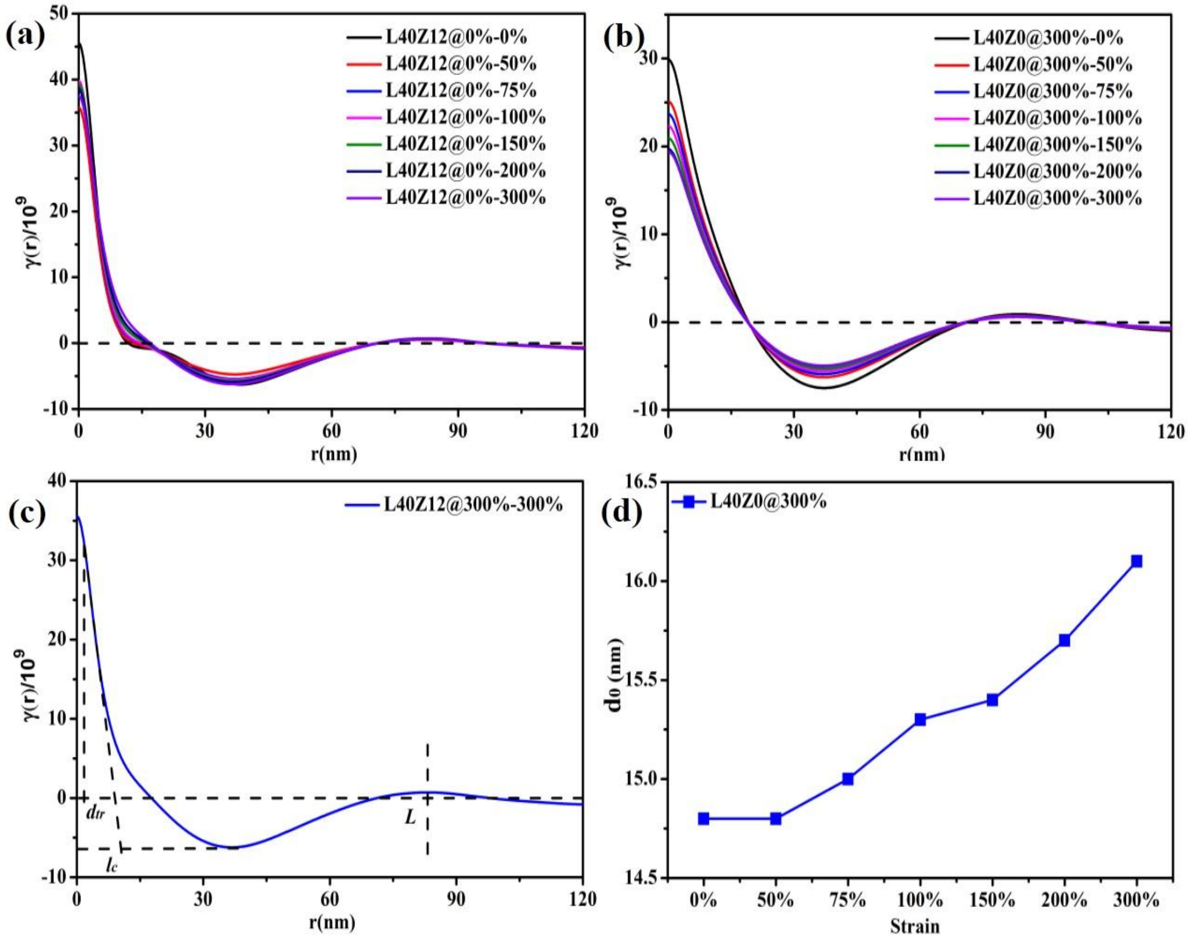

Supplementary Fig. 13 a) Normalized 1D correlation function curves for L40Z12@0% at various strains; b) Normalized 1D correlation function curves for L40Z0@300% at various strains; c) Normalized 1D correlation function curves for L40Z12@300% at 300% strain; d) The core-crystalline layer length (d_0) at different strains for L40Z0@300%.

Supplementary Table 1. Structural characteristics of L40Z12@300%, L40Z12@0% and L40Z0@300% at different tensile strains extracted from 1D correlation functions of SAXS curves.

Sample	Strain (%)	L (nm)	l_c (nm)	d_{tr} (nm)	$l_a = L\text{-}l_c$ (nm)	$d_0=l_c\text{-}d_{tr}$ (nm)	Δd_0 (nm)
L40Z12@300%	0	82.1	8.2	1.9	73.9	6.3	0
	50	83.5	8.6	2.0	74.9	6.6	0.3
	75	83.4	8.6	1.8	74.8	6.8	0.5
	100	82.4	8.7	1.9	73.7	6.8	0.5
	150	82.4	9.0	1.9	73.4	7.1	0.8
	200	83.2	9.7	1.8	73.5	7.9	1.6
	300	83.3	10.5	1.8	72.8	8.7	2.4
L40Z12@0%	0	82.6	8.2	2.2	74.4	5.9	0
	50	82.3	8.2	2.2	74.1	6.0	0.1
	75	83.1	8.5	2.1	74.6	6.2	0.3
	100	82.2	8.5	2.1	73.7	6.4	0.5
	150	82.5	8.9	2.0	73.6	6.9	1.0
	200	83.2	9.1	2.0	74.1	7.2	1.3
	300	82.7	9.8	2.1	72.9	7.7	1.8
L40Z0@300%	0	83.6	16.8	2.0	66.8	14.8	0
	50	83.3	16.8	2.0	66.6	14.8	0
	75	83.3	17.0	2.0	66.3	15.0	0.2
	100	82.9	17.3	2.0	65.6	15.3	0.5
	150	83.5	17.4	2.0	66.1	15.4	0.6

200	83.3	17.9	2.2	65.4	15.7	0.9
300	83.5	18.2	2.1	65.3	16.1	1.3

Δd_0: variation of d_0 before and after stretching

As shown in Supplementary Table 1, under the same strain, the d_0 and Δd_0 (variation of d_0 before and after stretching) of L40Z12@0% (without mechanical training) were both smaller than L40Z12@300%. While for L40Z0@300% without ZDMA, there was a higher initial d_0 since the crystalline region was not disturbed by ZDMA, but smaller growth ($\Delta d_0 = 1.3$ nm at 300% strain) compared with L40Z12@300% ($\Delta d_0 = 2.4$ nm at 300% strain) during the whole stretching process. These results further demonstrated that, after mechanical training, the sacrificial coordination bonds in the EPDM-based elastomer composite could not only stabilize chain orientation before stretching, but also promote the orientation of chain segments during the deformation course.

Explanation for the one-dimensional constitutive model

In order to demonstrate the variation of related network inside EPDM composite during the deformation course after mechanical training, a one-dimensional constitutive model is employed for describing the stress-strain relationship of materials. According to the characteristics of three-stage stress response, we assume that the molecular chain network is composed of three sub-networks, including covalent-bond network (σ_0), physical entangled network (σ_1) and coordination-bond network (σ_2) (**Fig.** 5a), and the corresponding rheological representations are illustrated as:

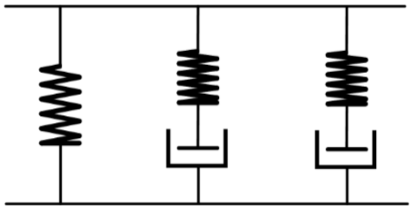

A permanent network

A physically entangled network

A dynamic coordination-bond network

By adopting Assumption that the total deformation of the three sub-networks is equal, $\lambda = \lambda_0 = \lambda_1 = \lambda_2$ are depicted as the total stretch.

Considering the inelastic deformation (viscous flow) of the physical network and the coordination-bond network, the total stretch is decomposed into an elastic part and a viscous part :

$$\lambda_1 = \lambda_1^e \lambda_1^v \quad (1)$$

$$\lambda_2 = \lambda_2^e \lambda_2^v \quad (2)$$

The total stress is decomposed into three parts:

$$\sigma = \sigma_0 + \sigma_1 + \sigma_2 \quad (3)$$

σ is the total nominal (engineering) stress.

Total true stress $\sigma_{true} = \lambda\sigma$ (4)

σ_0 corresponding to the stress of the permanent covalent-bond network

σ_1 corresponding to the stress of the physically entangled network

σ_2 corresponding to the stress of the dynamic coordination-bond network

By applying assumption that the material is incompressible, the permanent covalent-bond network (σ_0) is depicted by Carrillo-MacKintosh-Dobrynin (CMD) model:[5,6]

$$\sigma_0 = \frac{1}{\lambda}\frac{G_0}{3}\left(\lambda_0^2 - \lambda_0^{-1}\right)\left(1 + 2\left(1 - \frac{\beta_0\left(\lambda_0^2 + 2\lambda_0^{-1}\right)}{3}\right)^{-2}\right) \quad (5)$$

G_0 **is the shear modulus**.

β_0 **is the strand-extension ratio**. **This parameter determines the aligned degree of the polymeric strand between cross-links.**

β_0 **≡ $\langle R_{in}^2\rangle / R_{max}^2$** (6)**, $\langle R_{in}^2\rangle$ is the mean square end-to-end distance of undeformed network strands; R_{max}^2 is the square of the contour length of a fully extended strand.**

Similarity, the physically entangled network ($\sigma_1, I = 1$) and the dynamic coordination-bond network ($\sigma_2,\ I = 2$) are also depicted by (CMD) model.

$$\sigma_I = \frac{1}{\lambda}\frac{G_I}{3}\left(\lambda_I^{e2} - \lambda_I^{e-1}\right)\left(1 + 2\left(1 - \frac{\beta_I\left(\lambda_I^{e2} + 2\lambda_I^{e-1}\right)}{3}\right)^{-2}\right) \quad (7)$$

G_I **is the shear modulus.**

β_I **is the strand-extension ratio.**

β_I **≡ <R_{in}^2>/R_{max}^2** (8)

By adopting Bergström-Boyce (BB) model,[7,8] the viscous flow rate of the physically entangled network and dynamic coordination-bond network are depicted as:

$$\dot{\lambda}_I^v = \frac{1}{\sqrt{3}} \lambda_I^v sign(\sigma_I) \dot{\gamma}_0^v \left(\lambda_I^{eff} - 1 + \zeta\right)^{-1} \left(\frac{\tau_I^{eq}}{\overline{\tau}_I}\right)^{m_I} \quad (9)$$

where $\lambda_I^{eff} = \sqrt{\frac{1}{3}\left(\lambda_I^{v2} + 2\lambda_I^{v-1}\right)}$ is an effective stretch. (10)

$\tau_I^{eq} = \frac{\lambda \sigma_I}{\sqrt{3}}$ is an effective shear stress. (11)

$\overline{\tau}_I$ **is a material constant characterizing the strength of the polymer network**; m_I is a material constant characterizing the rate dependence of the stress, which is set as $m_I = 4$ in this work; $\dot{\gamma}_0^v = 1$ is a rate-dimensioned factor; ζ is the small positive constant for eliminating a removable singularity in the flow rate in the undeformed state, which is set as $\zeta = 0.004$ in this work.

Simulation results of the one-dimensional constitutive model

Supplementary Table 2. The related parameters of covalent-bond network (σ_0), physically entangled elastic network (σ_1) and coordination-bond network (σ_2) fitted from the engineering stress-strain curves of the composite samples mechanically trained at different training strain.

Sample	σ_0		σ_1			σ_2		
	G_0 (MPa)	β_0	G_1 (MPa)	β_1	$\bar{\tau}_1$ (MPa)	G_2 (MPa)	β_2	$\bar{\tau}_2$ (MPa)
L40Z12@200%	0.79	0.081	4	0.2	6	0.11	0.57	28
L40Z12@300%	0.75	0.105	4	0.2	6	0.15	0.49	54
L40Z12@400%	0.60	0.135	4	0.2	5	0.19	0.39	85
L40Z12@500%	0.58	0.170	4	0.2	5	0.18	0.38	102
L40Z12@600%	0.72	0.172	4	0.2	5	0.12	0.34	132

As shown in Supplementary Table 2, the parameters related to the physically entangled network (σ_1) were almost constant when the mechanical training strain varied, suggesting that the physically entangled network was elastic and reversible and not influenced by the mechanical training process. However, the parameters (β_0, β_2 and $\bar{\tau}_2$) related to the covalent-bond network (σ_0) and coordination-bond network (σ_2) obviously varied as the training strain increased from 200% to 600%. The strand-extension ratio, is the ratio of the mean square end-to-end distance of undeformed network strands $\langle R_{in}^2 \rangle$, to the square of the contour length of a fully extended strand R_{max} (Supplementary equation (6)). The value of β_0 for the covalent-bond network increased with the mechanical training strain, indicating that the chain segments between the covalent-bond crosslinks were more aligned (R_{in} ↑) and the covalent-bond

network was more easily to become taut as the training strain increased. The increasing value of β_0 could thus pull up the second-stage enhancement (Supplementary Fig. 14a). By contrast, the strand-extension ratio (β_2) of the coordination-bond network decreased with the training strain increased, conforming to the dynamic feature of coordination-bond network. With the training strain increased, more coordination bonds were not involved in regeneration course, followed by the increment of the contour length of a fully extended strand (R_{max} ↑), meaning that the coordination-bond network got looser, which would need larger strain to straighten out, thus the first-stage enhancement was postponed to a larger strain as the β_2 value decreased (Supplementary Fig. 14b, Supplementary equation (8)). Interestingly, although the number of coordination bonds declined as the training strain increased, the strength ($\bar{\tau}_2$) of the coordination-bond network was enhanced. The reason was that, as the training strain increased, more coordination bonds perpendicular to the direction of mechanical training were broken, followed by more coordination bonds reforming along the pre-stretched direction (**Figure** 5a), leading to that larger stress could be sustained in the first-stage enhancement as the $\bar{\tau}_2$ value increased (Supplementary Fig. 14c).

Based on the above simulation results, a facial strategy could be proposed for customizing the mechanical property of the artificial muscle material by precisely programming the input mechanical training strain, the density of coordination-bond network and covalent-bond network. Note that one merit of this strategy is that it does not require complex molecular design or sophisticated synthetic process.

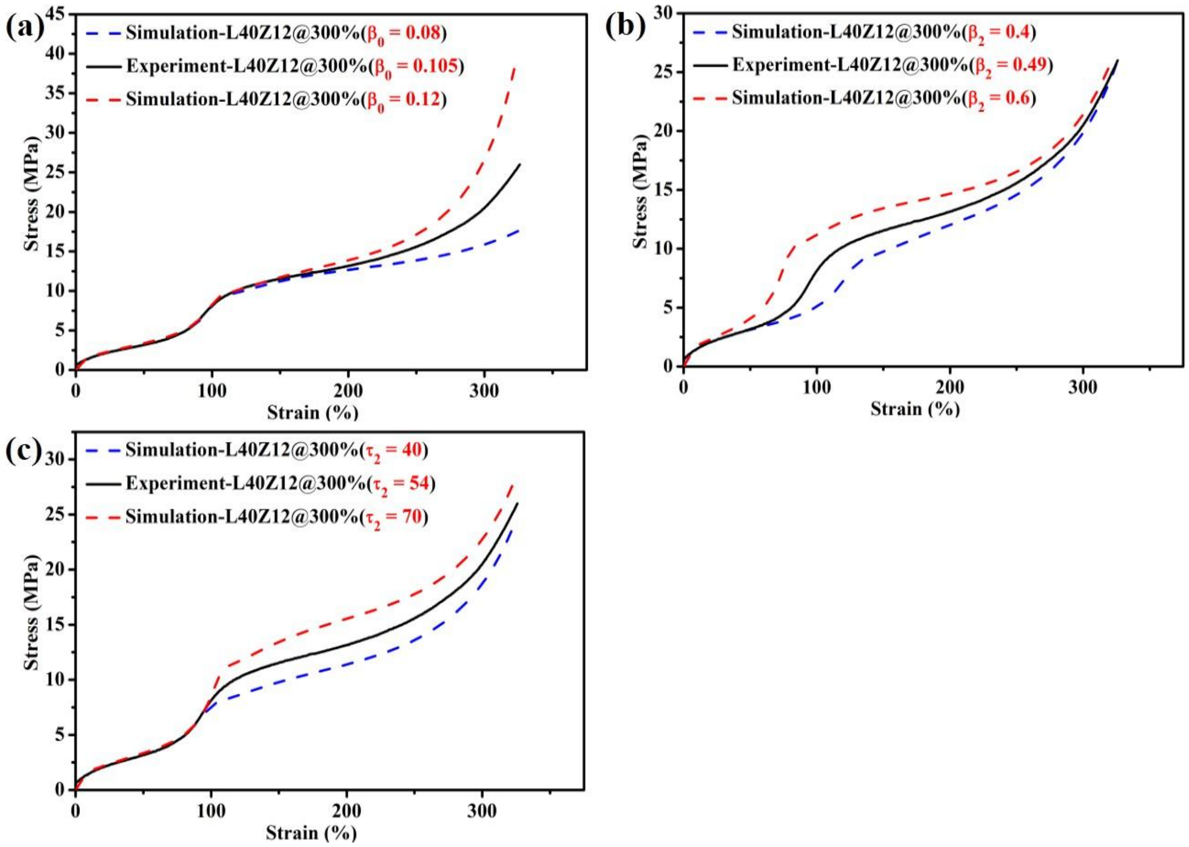

Supplementary Fig. 14 By mataining the other related structure parameters of L40Z12@300% unchanged, a) the simulation engineering stress-strain curves by adujusting the value of the value of β_0 ($\beta_2 = 0.49$; $\overline{\tau}_2 = 54$); b) The simulation engineering stress-strain curves by adujusting the value of the value of β_2 ($\beta_0 = 0.105$; $\overline{\tau}_2 = 54$); c) The simulation engineering stress-strain curves by adujusting the value of the value of $\overline{\tau}_2$ ($\beta_0 = 0.105$; $\beta_2 = 0.49$).

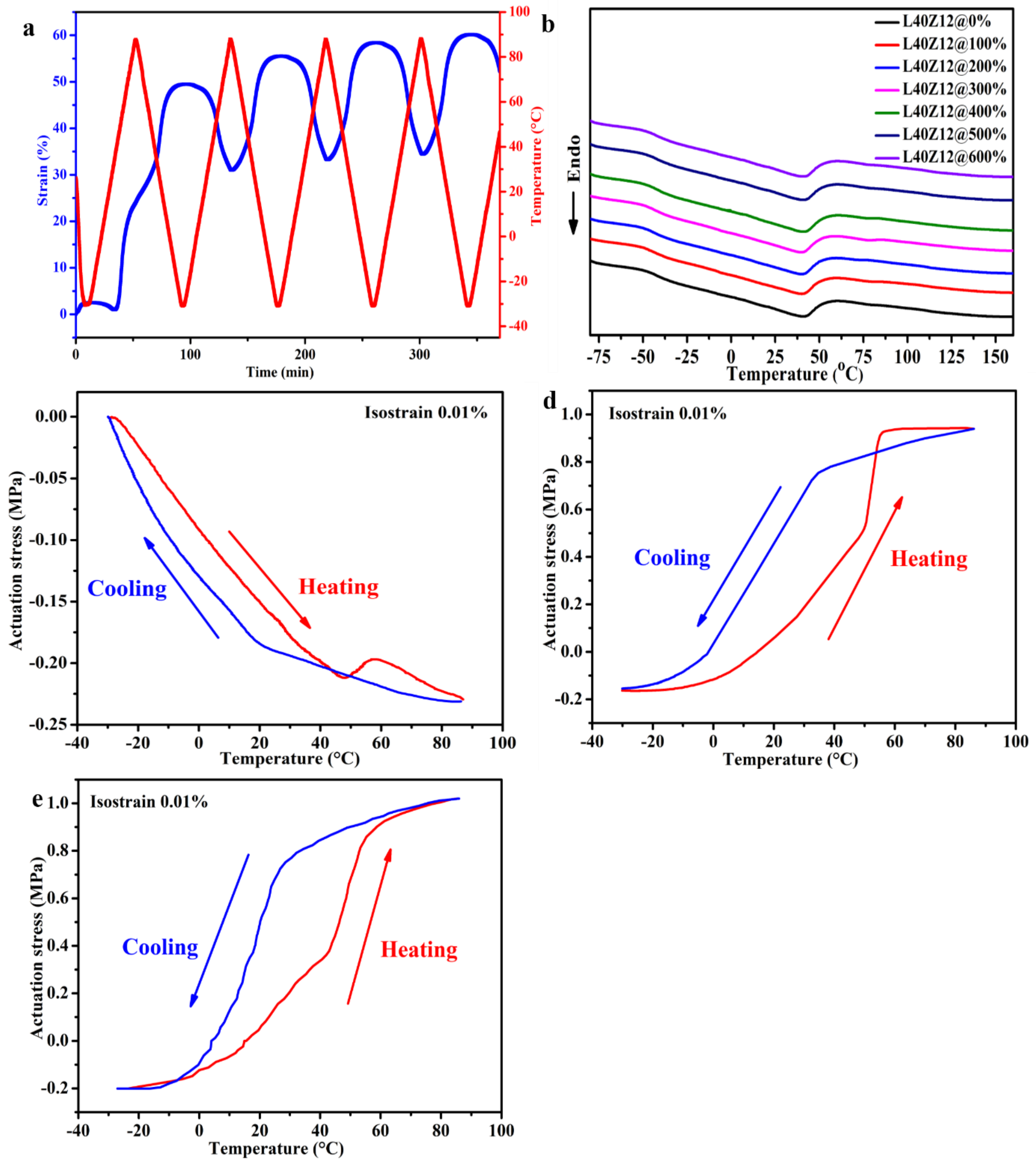

Supplementary Fig. 15 a)Strain variation in isoforce mode (1.0 MPa) and the corresponding temperature of the sample L40Z0@300% plotted against time; b) DSC crystallization curves of samples; c) Stress variation in isostrain mode of L40Z12@0% plotted against temperature; d) Stress variation in isostrain mode of L40Z0@300% plotted against temperature; e) Stress variation in isostrain mode of L0Z12@300% plotted against temperature.

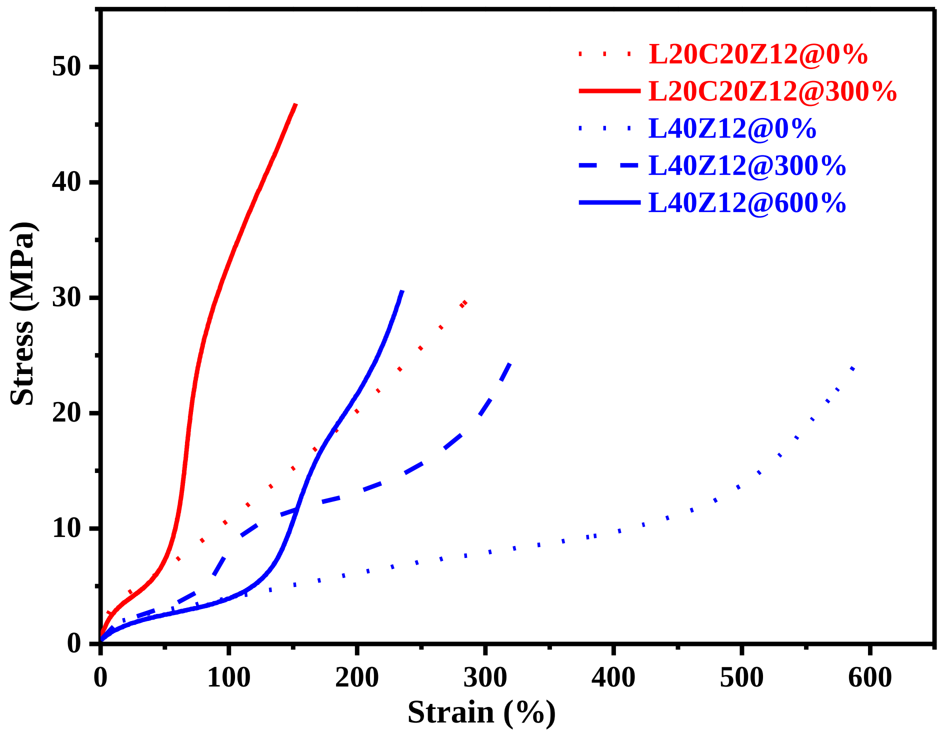

Supplementary Fig. 16 The engineering stress-strain curves of L20C20Z12@300% (Red) and L40Z12@300% and L40Z12@600% (Blue) after mechanical training for 250 cycles.

In order to study the electric programing performance, conductive carbon black was incorporated to obtain the electrical conductivity. However, when half percent of lignin was replaced by carbon black, the strain at break of the composite L20C20Z12@0% decreased to around 300% (Supplementary Fig. 16). To avoid the failure of the material, we chose 300% as the mechanical training strain. Larger percent of carbon black would lead to stiffer material and lose the characteristics of artificial muscle material, lower percent of carbon black could not achieve satisfactory conductivity. Thus, L20C20Z12@300% was selected to study the electric-induced actuation performance. The sample L20C20Z12@300% was processed by mechanical training at 300% for 250 pre-stretching cycles. As half mass of lignin was replaced by carbon black, the tensile strength of L20C20Z12@300% reached 47 MPa, the failure strain decreased to about 150%, the elastic modulus increased from 9 MPa to 100 MPa as the strain increased from around 50% to 75%, as shown in Supplementary Fig. 16. The self-strengthening effect and the strain-adaptive stiffening behavior of L20C20Z12@300%

were similar as L40Z12@600% and could mimic the mechanical performance of skeletal muscles.

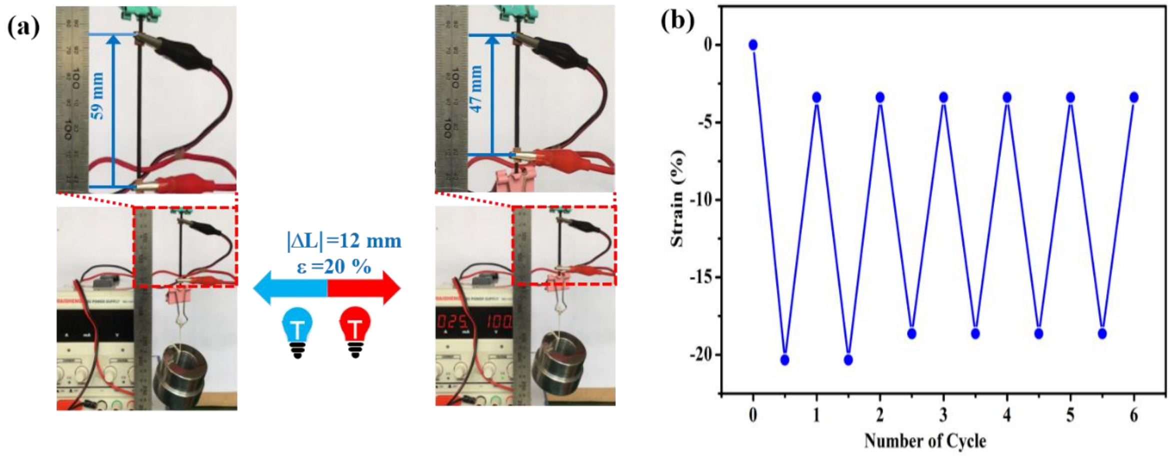

Supplementary Fig. 17 a) Photographs of the motion distance of L20C20Z12@300% under 0-25 mA (the color change of bulb represents the variation of the current); b) Plots of the reversible motion strain vs cycles for the thermal actuation of L20C20Z12@300% under 0-25 mA.

Reference

[1] Vatankhah-Varnosfaderani, M. et al. Chameleon-like elastomers with molecularly encoded strain-adaptive stiffening and coloration. *Science* **359**, 1509-1513 (2018).

[2] Sheiko, S. S. & Dobrynin, A. V. Architectural code for rubber elasticity: from supersoft to superfirm materials. *Macromolecules* **52**, 7531-7546 (2019).

[3] Vatankhah-Varnosfaderani, M. et al. Mimicking biological stress–strain behaviour with synthetic elastomers. *Nature* **549**, 497-501 (2017).

[4] Orza, R. A., Magusin, P. C. M. M., Litvinov, V. M., Van Duin, M., & Michels, M. A. J. Solid-state 1h nmr study on chemical cross-links, chain entanglements, and network heterogeneity in peroxide-cured epdm rubbers. *Macromolecules*, **40**, 8999-9008 (2007).

[5] Vatankhah-Varnosfaderani, M. et al. Mimicking biological stress–strain behaviour with synthetic elastomers. *Nature* **549**, 497-501 (2017).

[6] Carrillo, J-M. Y., Mackintosh, F. C. & Dobrynin, A. V. Nonlinear elasticity: from single chain to networks and gels. *Macromolecules* **46**, 3679-3692 (2013).

[7] Bergström, J. S. & Boyce, M. C. Constitutive modeling of the large strain time-dependent behavior of elastomers. *J. Mech. Phys. Solids.* **46**, 931-954 (1998).

[8] Bergstrom, J. S. *Mechanics of Solid Polymers: Theory and Computational Modeling*.(Matthew Deans, San Diego, 2015).
