# Supplementary material for: Biomimetic high performance artificial muscle built on sacrificial coordination network and mechanical training process
Source: Nat Commun. 2021 May 18;12:2916. doi: 10.1038/s41467-021-23204-x (PMC8131361; doi:10.1038/s41467-021-23204-x)
Supplement: Supplementary file 3 — Description of Additional Supplementary Files [file 41467_2021_23204_MOESM3_ESM.docx]

**Description of Additional Supplementary Files**

**Supplementary Movie 1:** Movie of the angle change vs cycles for the thermal actuation of L40Z12@600% lifting up a 200 g load;

**Supplementary Movie 2:** Movie of the length change vs cycles for the thermal actuation of L40Z12@600% (20 mg) bearing a load of 205 g which was 10000 times higher than its own weight;

**Supplementary Movie 3:** Movie of the length change of L20C20Z12@300% under varied current signals
